# Effectiveness of a Psychosocial Aftercare Program for Youth Aged 8 to 17 Years With Severe Chronic Pain: A Randomized Clinical Trial

**DOI:** 10.1001/jamanetworkopen.2021.27024

**Published:** 2021-09-27

**Authors:** Meltem Dogan, Gerrit Hirschfeld, Markus Blankenburg, Michael Frühwald, Rosemarie Ahnert, Sarah Braun, Ursula Marschall, Ingo Pfenning, Boris Zernikow, Julia Wager

**Affiliations:** 1 German Paediatric Pain Centre, Children’s and Adolescents’ Hospital, Datteln, Germany; 2 Department of Children’s Pain Therapy and Paediatric Palliative Care, Faculty of Health, Witten/Herdecke University School of Medicine, Witten, Germany; 3 Faculty of Business and Health, University of Applied Sciences Bielefeld, Bielefeld, Germany; 4 Paediatric Pain Center Baden-Württemberg, Department of Pediatric Neurology, Olgahospital Stuttgart, Stuttgart, Germany; 5 University Children’s Hospital Augsburg, Swabian Children’s Pain Center, Augsburg, Germany; 6 Department of Medicine and Health Services Research, BARMER Health Insurance, Wuppertal, Germany; 7 Techniker Krankenkasse, Techniker Health Insurance, Hamburg, Germany

## Abstract

**Importance:**

Severe chronic pediatric pain causes individual suffering and significantly affects social functioning and psychological well-being. For children with high pain severity, intensive interdisciplinary pain treatment (IIPT) is a well-established treatment. However, across specialized centers, it is not sufficient for all patients.

**Objective:**

To evaluate the effectiveness of a psychosocial aftercare (PAC) program for pediatric patients with severe chronic pain followed up for 6 months after discharge from IIPT.

**Design, Setting, and Participants:**

This multicenter randomized clinical trial with 4 assessment points (pre-IIPT, immediately post-IIPT, 3 months, and 6 months) was conducted at 3 pediatric specialized tertiary care pain centers in Germany between September 11, 2018, and March 31, 2020. Included patients were aged 8 to 17 with a severe chronic pain condition who had been admitted for IIPT. Data were analyzed from June 8 to September 4, 2020.

**Interventions:**

Patients and their families were randomly assigned to 1 of 2 study groups at inpatient IIPT admission. Both groups received standardized 3- to 4-week IIPT. After IIPT discharge, the intervention group received PAC and the control group received usual care. PAC involved ongoing contact with a social worker for as long as the family requested the support, up to a maximum of 6 months.

**Main Outcomes and Measures:**

The primary outcome measure was pain at 6 months, measured using the Chronic Pain Grading (CPG), an instrument based on an algorithm indicating severity of the chronic pain disorder. Secondary outcomes included other pain-related and emotional parameters.

**Results:**

A total of 419 patients were randomized (mean [SD] age, 14.3 [2.1] years; 303 [72.3%] girls; 116 [27.7%] boys), with 218 assigned to usual care and 201 assigned to PAC. At baseline in both groups, the median (IQR) CPG was 3 (2-4). Superiority of PAC compared with usual care was demonstrated at 6 months (median [IQR] CPG: usual care, 2 [2-3]; PAC, 1 [1-2]; *r* = 0.30; 95% CI, 0.17-0.41). Additionally, PAC significantly improved emotional parameters (eg, significant time × group interaction: *b* = −8.84; *P* < .001).

**Conclusions and relevance:**

This randomized clinical trial found that PAC improved pain-related and emotional parameters during the intervention 6 months after discharge from IIPT. Future research is needed to investigate the intervention’s long-term effects.

**Trial Registration:**

German Clinical Trials Register ID: DRKS00015230

## Key Points

**Question:**

Does a psychosocial aftercare program (PAC) after in-hospital intensive interdisciplinary pain treatment (IIPT) for pediatric patients with chronic pain improve functional outcomes?

**Findings:**

In this randomized clinical trial including 419 patients aged 8 to 17 years randomized and 222 patients analyzed at 6 months, PAC significantly improved all pain-related as well as emotional outcome parameters compared with treatment as usual.

**Meaning:**

These findings suggest PAC should be considered in IIPT for pediatric chronic pain and its effectiveness tested in other pediatric psychosomatic conditions.

## Teaser

This randomized clinical trial evaluates the effectiveness of a psychosocial aftercare program for pediatric patients with severe chronic pain vs usual care after discharge from intensive interdisciplinary pain treatment.

## Introduction

Intensive interdisciplinary pain treatment (IIPT) is a well-established treatment for severe pediatric chronic pain.^1^ While this specialized treatment demonstrates long-term effectiveness for many patients, some do not benefit sufficiently.^2,3^ In the absence of empirical data, clinical observations indicate a plausible source of risk for treatment failure: after a short intensive treatment of 3 to 4 weeks,^1^ patients may have difficulty generalizing and maintaining individual therapy goals during the transition from highly structured, clinician-led support to self-reliance in everyday life. For example, patients with psychological comorbidities often do not engage with a psychotherapist after IIPT, despite this being recommended.^4^ The transtheoretical model^5^ highlights that changing maladaptive health behaviors can be challenging. Consolidating and generalizing the active behavior learnt during treatment requires a substantial change in coping efforts. Interventions supporting transition from clinician-led treatment to self-determined use of learned strategies have been assessed in other chronic health conditions. Meta-analyses provide evidence that clinician-led multicomponent interventions (eg, parent education or behavioral training) can empower pediatric patients with chronic health conditions, such as asthma or diabetes, to self-reliantly follow their treatment plan, leading to improvements in disease management and disease severity.^6^ In light of this evidence, an adjunctive family-centered treatment module for severe chronic pain was developed: personalized psychosocial aftercare (PAC), which aims to provide support for pediatric patients and their families in adherence to discharge recommendations and facilitate maintenance of IIPT treatment outcomes.

To test the effectiveness of PAC as an adjunctive IIPT treatment module, we conducted a multicenter randomized clinical trial comparing treatment effectiveness in patients receiving PAC compared with patients receiving usual aftercare. A real-world study design was chosen to compare these aftercare strategies in a typical clinical setting. We expected that PAC would lead to greater improvements in pain severity (incorporating pain intensity and functional impairment) and other pain-related and psychological outcomes compared with usual care 3 and 6 months after discharge. Moreover, we expected that at both time points, patients who received PAC would show greater overall therapy satisfaction and greater adherence to therapy recommendations than patients who received usual care.

## Methods

This randomized clinical trial was approved by the ethics committees of Witten/Herdecke University, Baden-Wuerttemberg State Chamber for Medicine, and the Faculty of Medicine at Ludwig Maximilian University Munich. Written informed consent was obtained from all participants’ parents, and all participants provided written informed assent. This study is reported following the Consolidated Standards of Reporting Trials (CONSORT) reporting guideline.

### Study Design

The study was planned as a 2-arm parallel samples prospective multicenter randomized clinical trial with 5 assessment points in a realistic clinical setting (Trial Protocol in Supplement 1). The originally planned assessment points included: pre-IIPT (IIPT admission), post-IIPT (IIPT discharge), 2 assessments during the intervention period (at 3 and 6 months), and a follow-up assessment 12 months after discharge from IIPT. As a result of the COVID-19 pandemic and the consequential school closings in Germany from March 18, 2020, onwards, the last assessment point was not stopped but excluded from these analyses because school absence, a core component of Chronic Pain Grading (CPG), could no longer be reliably assessed.

For randomization, one of us (G.H.) created computer-generated randomization lists. Patients were randomly assigned to 1 of 2 study groups: PAC or usual care. Blinding was not feasible. Furthermore, the study design embodied typical characteristics of real-world trials,^7^ including the application of few exclusion criteria, monitoring of (but not dictating) intervention frequency and intensity, and a primary outcome measure consisting of aggregated patient-reported symptoms of high importance to patients.

### Study Sample

**Figure 1.**
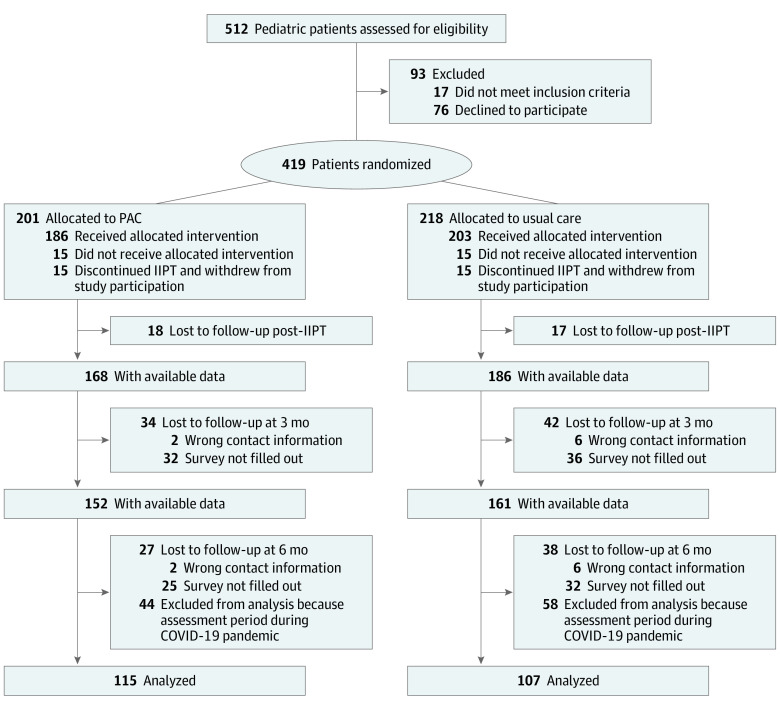
Study Participant Flowchart IIPT indicates intensive interdisciplinary pain treatment; PAC, psychosocial aftercare program.

Inclusion criteria were pediatric patients aged 8 to 17 years admitted to IIPT from September 2018 to October 2019, patient’s and at least 1 parent’s sufficient German language skills to complete questionnaires and engage in the treatment (determined by clinician or nurse judgment), and patient’s and parent’s agreement to study participation. In Germany, children with chronic pain are eligible for IIPT if they fulfil at least 3 of the following criteria: reduced quality of life owing to pain, unsuccessful unimodal pain treatment, medication misuse, presence of psychological comorbidity, and presence of somatic comorbidity.^8^ Most of these patients are diagnosed with either *International Statistical Classification of Diseases and Related Health Problems, Tenth Revision, German Modification* (*ICD-10-GM*) code F45.40 or F45.41. The only exclusion criterion was withdrawal of study participation. The sample size was determined a priori based on anticipated between-group differences of the primary outcome of pain severity. Assuming an effect size of *d* = 0.5, an α level of 5%, and a power of 95%, we calculated that 92 patients per group were needed to detect an advantage of PAC using a 1-sided Mann-Whitney test. Based on attrition rates of approximately 50% reported by prior studies with comparable follow-ups, 419 patients were recruited to achieve the desired sample size at follow-up. Figure 1 displays the participant recruitment flowchart.

### Procedure

At each participating center, patients were assessed for eligibility immediately after IIPT admission. Patients meeting the inclusion criteria were informed about the study and randomized after a least 1 parent’s written informed consent and the patient’s informed assent. Patients then completed the first set of study questionnaires on an electronic tablet (pre-IIPT). The second set was completed on discharge day (post-IIPT). For the following assessments, survey links were sent via email. All participants received manualized IIPT^9^ (eAppendix 1 in Supplement 2). After discharge, the intervention group received PAC while the control group received usual care.

### Control Group: Usual Care

Patients in the control group received the standard aftercare after IIPT. This involved the option of a 1- to 1.5-hour refresher session with the treating pediatrician and psychotherapist 3 and 6 months after discharge at the pain center (eAppendix 1 in Supplement 2). Additional sessions were optional and by request.

### Intervention Group: PAC

Patients in the intervention group were offered the same standard aftercare as the control group. In addition, patients received personalized psychosocial aftercare (PAC). This intervention was based on a standardized manual and guided by the concept of case-management. A detailed description of PAC is provided in eAppendix 2, eTable 1, and the eFigure in Supplement 2.

### Measures

#### Patient Characteristics

Patient and pain characteristics (eg, demographics, pain location and duration) were assessed by the German Pain Questionnaire for Children and Adolescents.^10^ This semi-structured instrument assesses multiple aspects of the child's pain experience.

#### Primary Outcome

The primary outcome, pain severity, was derived using the Chronic Pain Grading (CPG),^7^ an instrument to classify chronic pain patients into different severity grades (ranging from 0-4, with 0, indicating no chronic pain; 1, low pain intensity and low disability; 2, high pain intensity and low disability; 3, high disability, moderately limiting; and 4, high disability, severely limiting).^11^ Chronic pain severity is calculated based on an algorithm including the patient’s rating of pain intensity (overall mean of maximum rating and mean rating over the past 4 weeks), days absent from school, and pain-related disability in everyday life^11^ (eAppendix 3 in Supplement 2). Maximum and mean pain intensity within the past 4 weeks were reported on a numerical rating scale (NRS) (range, 0-10, with 0 indicating no pain and 10, strongest pain).^12^ Patients reported the number of days they had missed school owing to pain within the past 4 weeks (maximum: 20 days). Pain-related disability was measured using the validated Paediatric Pain Disability Inventory (PPDI),^13,14^ which consists of 12 self-report items assessing pain-related disability in daily activities, eg, reading or sports, on a Likert scale (range, 1-5, with 1 indicating never and 5, always; total score, 12-60, with higher values indicate greater disability; internal consistency in this sample: Cronbach α = .87). The assessment of missed school days and pain-related disability was not assessable at post-IIPT; thus, CPG was not calculable at that point.

#### Secondary Outcomes

Secondary outcomes included pain-related characteristics, psychological measures, and treatment-related measures. Pain intensity, pain-related school absence and pain-related disability were included as pain-related characteristics.

Pain self-efficacy, a measure of one’s confidence in successfully dealing with pain by implementing pain coping strategies,^15^ was measured with the validated Scale for Pain Self-Efficacy. This tool consists of 11 items (eg, “Even when I am in pain, I can still do the things I like doing”), with responses reported on a Likert scale (range, 0-4, with 0 indicating not true and 4, true; higher values indicate greater self-efficacy). This scale has good internal consistency (in this sample: Cronbach α = .86).

Depression and anxiety symptoms were assessed using the validated Revised Child Anxiety and Depression Scale,^16,17^ a 47-item self-reported measure with a depression and an anxiety subscale (in this sample: Cronbach α depression = .87; Cronbach α anxiety = .89). The items are reported on a Likert scale (range, 0-3, with 0 indicating never to 3, always; higher values indicate greater depression and anxiety symptoms).

Health-related quality of life was measured using the validated Kidscreen-27 (in this sample: Cronbach α = .91).^18^ This measure consists of 5 dimensions (physical well-being, psychological well-being, autonomy and parents, peers and social support, and school environment) summing up in a total score. Items are rated on Likert scales (range, 0-5, with 1 indicating never and 5, always; higher values indicate greater quality of life). This tool was not used at post-IIPT.

During PAC treatment, therapy adherence was reported at 3 and 6 months by patients on an NRS (range, 0-10, with 0 indicating not at all to 10, completely agree) using the items “I was able to implement the recommendations from IIPT” and “It was easy to implement the recommendations from IIPT.” Additionally, PAC intensity (defined as the duration of all therapeutic contacts) and PAC frequency (defined as the number of treatment contacts) were documented by social workers for the whole duration of intervention.

At post-IIPT and during PAC-treatment, overall treatment satisfaction was reported by patients on an NRS using the item “I am satisfied with my overall pain treatment.”

### Statistical Analysis

All statistical analyses were conducted by 1 of us (G.H.) according to the approach defined in the statistical analysis plan (Supplement 1). First, we examined whether study groups differed in demographic and pain-related variables at pre-IIPT (continuous variables: *t* tests and Welch tests; categorical variables: χ^2^ tests). Second, changes in the primary outcome (CPG, ordinal data) from pre-IIPT to 3 and 6 months were calculated with the Wilcoxon test. Pain outcomes at 3 and 6 months were compared with pre-IIPT scores because disability scores were not calculable at post-IIPT. We then used the Mann-Whitney *U* test to compare the distribution of the primary outcome (CPG) between groups at 3 and 6 months. Additionally, in the PAC group alone, Spearman correlations between CPG improvement and PAC intensity and PAC frequency were assessed. Third, the secondary outcomes (continuous data) were analyzed using mixed-model analyses (eAppendix 3 in Supplement 2). Last, treatment satisfaction, adherence with treatment recommendations, and perceived ease of adherence was compared between groups using *t* tests. Results of sensitivity analyses using all available data and multiple imputation are described in eAppendix 4 in Supplement 2.

We used R statistical software version 4.0.3 (R Project for Statistical Computing) for our analyses. *P* values were 1-sided for our primary outcome and 2-sided for all secondary outcomes. Statistical significance was set at *P* = .05. Data were analyzed from June 8 to September 4, 2020.

## Results

### Patient Characteristics

A total of 419 pediatric patients with chronic pain (mean [SD] age, 14.3 [2.1] years; 303 [72.3%] girls; 116 [27.7%] boys) were randomized, with 218 patients assigned to usual care and 201 assigned to PAC. Table 1 provides an overview of demographic and clinical characteristics of the study sample and treatment groups. At pre-IIPT, the groups did not differ significantly in sociodemographic or pain-related characteristics, and the median (IQR) CPG for both groups was 3 (2-4). A total of 102 (24.3%) patients were excluded from analyses because they supplied 6-month follow-up data after the pandemic-related school-closings in Germany, and 95 patients (22.7%) dropped out of analyses for other reasons (Figure 1). No systematic dropout was detected, as patients who completed the assessment at 6 months (222 patients, including 107 patients in the usual care group and 115 patients in the PAC group) did not differ in pre-IIPT-characteristics from patients who were excluded from analyses owing to the COVID-19 pandemic, or patients who dropped out for other reasons (eTable 2 in Supplement 2).

**Table 1.** Sociodemographic and Pain-Related Characteristics at Study Inclusion

Characteristic	Patients, No. (%)		
	All (N = 419)	Usual care (n = 218)	PAC (n = 201)
Sex			
Girls	303 (72.3)	155 (71.1)	148 (73.6)
Boys	116 (27.7)	63 (28.9)	53 (26.4)
Age, mean (SD), y	14.3 (2.1)	14.3 (2.05)	14.3 (2.16)
Country of birth			
Germany	406 (96.9)	211 (96.8)	195 (97.0)
Syria	1 (0.2)	1 (0.5)	0
Russia	1 (0.2)	0	1 (0.5)
Poland	2 (0.5)	1 (0.5)	1 (0.5)
Other	9 (2.2)	5 (2.2)	4 (2.0)
Pain locations^a^			
Head	288 (68.7)	150 (68.8)	138 (68.7)
Abdomen	109 (26.0)	55 (25.2)	54 (26.9)
Musculoskeletal	183 (43.7)	89 (40.8)	94 (46.8)
>1 main pain location	132 (31.5)	61 (28.0)	71 (35.4)
Pain duration			
3-6 mo	37 (8.83)	19 (8.72)	18 (8.96)
6-12 mo	85 (20.3)	42 (19.3)	43 (21.4)
1-2 y	69 (16.5)	32 (14.7)	37 (18.4)
2-3 y	79 (18.9)	48 (22.0)	31 (15.4)
>3 y	149 (35.6)	77 (35.3)	72 (35.8)
Pain intensity, mean (SD)^b^			
Maximum, mean (SD)	8.15 (1.74)	8.08 (1.88)	8.22 (1.58)
Overall	6.07 (1.89)	6.01 (1.95)	6.12 (1.84)
Missed school, mean (SD), d^b^	5.44 (6.93)	5.24 (6.79)	5.66 (7.10)
CPG score^c^			
1	31 (7.4)	16 (7.3)	15 (7.5)
2	137 (32.7)	76 (34.9)	61 (30.3)
3	137 (32.7)	71 (32.6)	66 (32.8)
4	114 (27.2)	55 (25.2)	59 (29.4)

Abbreviations: CPG, Chronic Pain Grading; PAC, psychosocial aftercare program.

^a^ Multiple pain locations could be selected.

^b^ In the past 4 weeks.

^c^ At admission no participants had a CPG score of 0.

### Primary Outcome

In both treatment groups at 3 months, as well as at 6 months, the primary outcome CPG (pain severity) showed significant improvements compared with pre-IIPT (usual care: 3 months, *r* = 0.36; 95% CI, 0.17-0.41; *P* < .001; 6 months, *r* = 0.42; 95% CI, 0.26-0.57; *P* < .001; PAC: 3 months, *r* = 0.65; 95% CI, 0.53-0.74; *P* < .001; 6 months, *r* = 0.75; 95% CI, 0.65-0.80; *P* < .001). The CPG differences between usual care and PAC groups showed a moderate effect at 6 months (median [IQR] CPG: usual care, 2 [2-3]; PAC, 1 [1-2]; *r* = 0.30; 95% CI, 0.17-0.41; *P* < .001). Specifically, 25 patients (23.3%) in the usual care group had reached a CPG 0 or 1, compared with 58 patients (50.4%) in the PAC group. Both groups had a comparable percentage of patients in CPG 2 (usual care: 43 patients [40.2%]; PAC: 41 patients [35.7%]). The usual care group had a higher proportion of participants with CPG 3 or 4 (39 patients [36.4%]) than the PAC-group (16 patients [13.0%]). A detailed description of CPG distribution is provided in eTable 3 in Supplement 2. Exploratory analyses also showed a difference between groups at 3 months (*r* = 0.17; 95% CI, 0.06-0.28; *P* = .008). No significant correlations were identified between CPG improvement at 6 months and PAC intensity (Spearman ρ = .15; *P* = .52) or PAC frequency (Spearman ρ = .14; *P* = .64), indicating these measures were not linearly associated with CPG improvement. The CPG distribution for all available data can be found in eTable 4 in Supplement 2.

### Secondary Outcomes

Means and mean differences of all secondary outcomes for each group and across time points are in Table 2. Mixed-model analyses were conducted to determine whether differences existed by time point, group, or time-by-group interaction for secondary pain and psychological outcomes.

**Table 2.** Raw Means and Mean Differences of Secondary Outcomes

Outcome	Pre-IIPT		Post-IIPT		3 mo		6 mo	
	Usual care (n = 107)	PAC (n = 115)	Usual care (n = 102)	PAC (n = 108)	Usual care (n = 99)	PAC (n = 108)	Usual care (n = 107)	PAC (n = 115)
**Pain measures**								
Maximum pain intensity (last 4 wk), mean (SD)^a^	8.10 (1.92)	8.23 (1.56)	7.78 (2.13)	7.80 (2.26)	7.33 (2.83)	6.33 (3.01)	6.86 (2.84)	5.34 (3.32)
Mean difference (95% CI)	−0.13 (−0.57 to 0.31)		−0.01 (−0.58 to 0.56)		1.00 (0.21 to 1.79)		1.52 (0.71 to 2.34)	
Pain intensity (last 4 wk), mean (SD)^a^	6.20 (1.98)	6.08 (1.83)	5.00 (2.17)	4.75 (2.34)	5.24 (2.71)	4.24 (2.51)	5.25 (2.80)	3.63 (2.88)
Mean difference (95% CI)	−0.12 (−0.37 to 0.60)		0.25 (−0.24 to 0.84)		1.00 (0.27 to 1.73)		1.62 (0.87 to 2.37)	
Missed school (last 4 wk), mean (SD), d	5.37 (6.65)	5.94 (7.12)	NA	NA	2.86 (4.78)	2.53 (4.97)	2.71 (4.63)	1.32 (3.79)
Mean difference (95% CI)	−0.57 (−2.38 to 1.25)		NA		0.34 (−1.17 to 1.84)		1.39 (0.16 to 2.63)	
Pain-related disability, mean (SD)^b^	34.95 (9.76)	36.05 (9.64)	NA	NA	29.48 (13.42)	26.00 (12.23)	29.50 (12.70)	22.90 (11.10)
Mean difference (95% CI)	−1.10 (−3.60 to 1.40)		NA		3.48 (0.02 to 6.95)		6.59 (3.43 to 9.75)	
**Psychological measures**								
Pain self-efficacy, mean (SD)^c^	26.88 (8.43)	27.56 (9.23)	37.01 (9.37)	37.62 (9.26)	33.31 (11.54)	38.10 (9.21)	33.34 (12.10)	40.62 (9.82)
Mean difference (95% CI)	−0.68 (−2.97 to 1.62)		−0.61 (−3.13 to 1.91)		−4.80 (−7.78 to −1.81)		−7.28 (−10.41 to −4.16)	
Depression, mean (SD)^d^	10.84 (5.43)	11.58 (5.62)	8.77 (5.21)	9.65 (5.51)	11.10 (5.64)	9.23 (5.65)	10.84 (5.79)	8.42 (5.32)
Mean difference (95% CI)	−0.74 (−2.25 to 0.77)		−0.88 (−2.40 to 0.65)		1.87 (0.26 to 3.48)		2.42 (0.95 to 3.9)	
Anxiety, mean (SD)^d^	26.94 (17.83)	29.74 (18.53)	24.35 (17.13)	29.08 (17.72)	26.64 (17.52)	23.22 (18.34)	25.51 (18.63)	19.90 (16.72)
Mean difference (95% CI)	−2.80 (−7.52 to 1.93)		−4.73 (−9.84 to 0.38)		3.41 (−1.78 to 8.60)		5.62 (0.93 to 10.3)	
Health-related quality of life, mean (SD)^e^	96.2 (14.91)	95.5 (15.68)	NA	NA	95.5 (17.31)	103.1 (16.23)	96.2 (14.91)	95.5 (15.68)
Mean difference (95% CI)	0.66 (−3.27 to 4.59)		NA		−7.59 (−12.09 to −3.08)		−10.79 (−15.18 to −6.40)	

Abbreviations: IIPT, intensive interdisciplinary pain treatment; NA, not applicable; PAC, psychosocial aftercare program.

^a^ Scored on a numerical rating scale (range, 0-10; higher scores indicate worse pain).

^b^ Scored using the Paediatric Pain Disability Index (range, 12-60; higher scores indicate more disability).

^c^ Scored using the Scale for Pain Self-Efficacy (range, 0-44; high score indicates more self-efficacy).

^d^ Scored using the Revised Child Anxiety and Depression Scale (depression: range 0-30; anxiety: range, 0-111; higher scores indicate worse depression or anxiety).

^e^ Scored using Kidscreen-27 (range: 27-135; higher scores indicate better quality of life).

**Figure 2.**
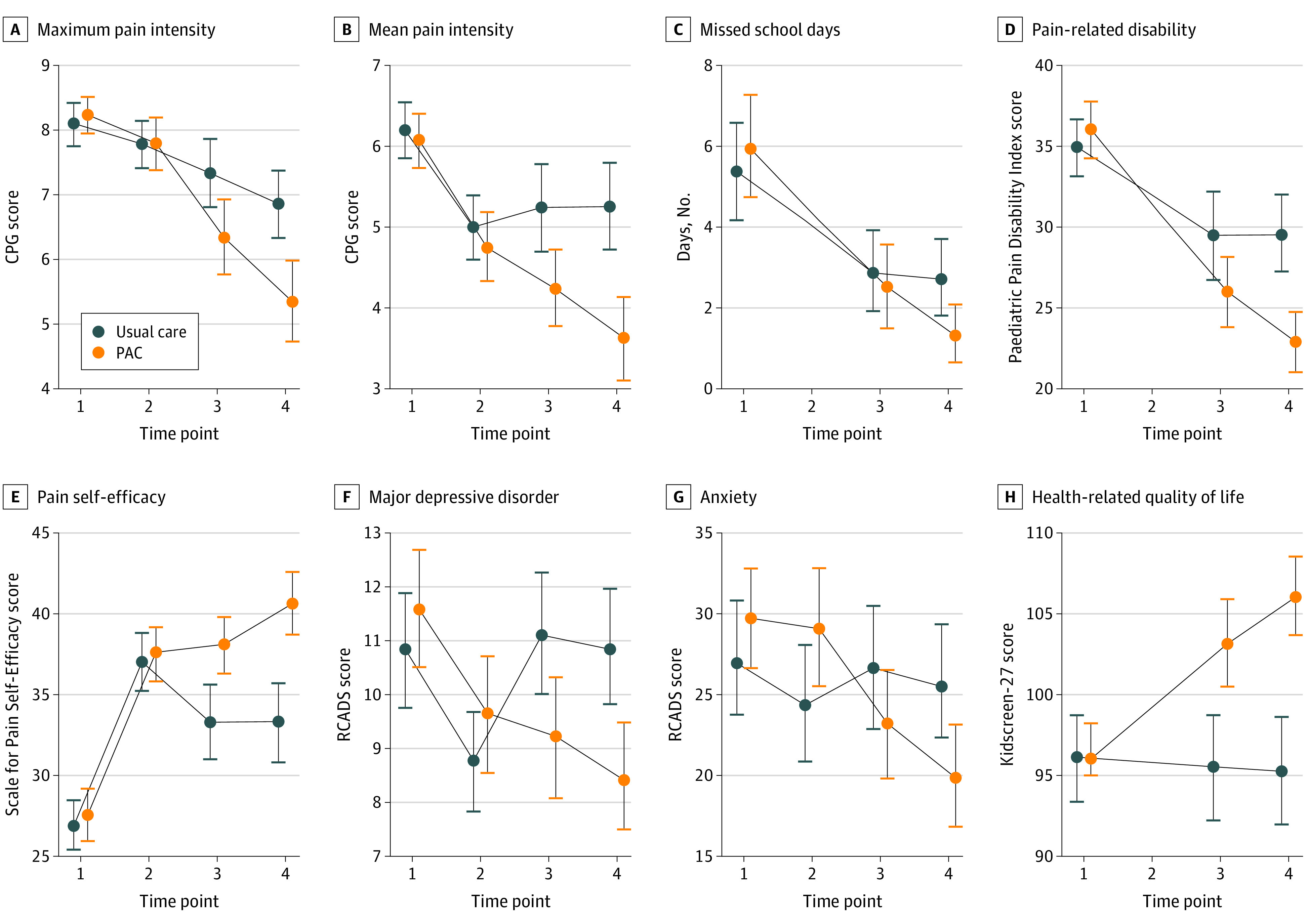
Descriptive Statistics of the Secondary Outcomes at All Assessment Time Points for Both Groups Missed school days and pain-related disability were not assessable at time point 2. Time points are defined as 1, pre–intensive interdisciplinary pain treatment (IIPT); 2, post-IIPT; 3, 3 months post-IIPT; and 4, 6 months post-IIPT. CPG indicates Chronic Pain Grading; RCADS, Revised Child Anxiety and Depression Scale.

As shown in Figure 2, all pain characteristics improved over time in both groups, but the improvements were greater for the PAC-group compared with the usual care group. Mixed-model analyses revealed a main effect of time for maximum and mean pain intensity (Table 3). Moreover, there were significant interactions between time and group at 3 months and 6 months, indicating that the improvement was greater in the PAC group than in the usual care group.

**Table 3.** Mixed-Model Analysis Results

	Coefficient (95% CI)^a^							
Measure	Group	Time (post-IIPT)	Time (post-IIPT) × group	Time (3-mo)	Time (3-mo) × group	Time (6-mo)	Time (6-mo) × group
Maximum pain intensity	0.15 (−0.51 to 0.81)	−0.25 (−0.83 to 0.32)	−0.12 (−0.84 to 0.60)	−0.76 (−1.35 to −0.16)	−1.18 (−2.00 to −0.36)	−1.22 (−1.83 to −0.62)	−1.68 (−2.53 to −0.84)
Mean pain intensity	−0.12 (−0.75 to 0.52)	−1.19 (−1.66 to −0.73)	−0.15 (−0.95 to 0.65)	−0.95 (−1.50 to −0.41)	−0.92 (−1.67 to −0.16)	−0.96 (−1.52 to −0.39)	−1.49 (−2.28 to −0.70)
Missed school	0.58 (−1.14 to 2.31)	NA	NA	−2.43 (−3.57 to −1.30)	−0.89 (−2.48 to 0.69)	−2.92 (−4.26 to −1.57)	−1.83 (−3.71 to 0.06)
Pain-related disability	1.06 (−1.90 to 4.03)	NA	NA	−5.75 (−7.85 to −3.65)	−4.52 (−7.44 to −1.61)	−5.49 (−7.99 to −2.98)	−7.63 (−11.12 to −4.14)
Pain self-efficacy	0.76 (−1.83 to 3.34)	9.94 (8.05 to 11.83)	0.12 (−2.51 to 2.75)	6.62 (4.35 to 8.89)	4.19 (1.04 to 7.35)	6.75 (4.24 to 9.25)	6.36 (3.05 to 9.68)
Depression	0.82 (−0.93 to 2.57)	−1.86 (−2.81 to −0.91)	0.03 (−1.29 to 1.35)	0.23 (−0.89 to 1.34)	−2.62 (−4.17 to −1.08)	0.03 (−1.13 to 1.18)	−3.17 (−4.78 to −1.57)
Anxiety	3.01 (−1.79 to 7.80)	−1.83 (−4.43 to 0.77)	1.03 (−2.59 to 4.65)	−0.71 (−3−93 to −2.51)	−6.02 (−10.48 to −1.57)	−0.98 (−5.15 to 3.19)	−8.84 (−13.66 to −4.02)
Health-related quality of life	−0.71 (−4.90 to 3.47)	NA	NA	−0.23 (−3.23 to −2.78)	8.13 (3.96 to 12.30)	−0.93 (−4.30 to 2.45)	11.60 (6.90 to 16.30)

Abbreviations: IIPT, intensive interdisciplinary pain treatment; NA, not applicable.

^a^ The reference category for time was pre-IIPT.

Regarding school absence, in addition to the main effect of time, the interaction coefficients showed no significant difference between groups. For pain-related disability, there was a main effect of time as well as greater reductions in in the PAC group than in the usual care group at 3 months and 6 months, as indicated by the significant time-by-group interaction.

In addition to a main effect of time, analyses of self-efficacy identified that trajectories diverged at 3 and 6 months, per significant time-by-group interaction terms (Figure 2 and Table 3). While self-efficacy increased at both time points in the PAC group, it decreased in the usual care group.

Main effects of time for depression and anxiety were only found at post-IIPT but not at 3 and 6 months. Significant time-by-group interactions emerged at 3 months and 6 months, because depressive and anxiety symptoms returned to admission levels in the usual care group, while they further decreased in the PAC group (Table 3).

Health-related quality of life showed no overall improvement over time. Significant time-by-group interactions emerged at 3 months and 6 months owing to increasing quality of life in the PAC group and stable values in the usual care group (Table 3).

Overall treatment satisfaction was high at post-IIPT, with groups not significantly differing regarding this measure. However, patients in the PAC group reported a higher satisfaction at 6 months (change, −1.18; 95% CI, −1.90 to −0.045; *P* = .002) compared with the usual care group. At 6 months, the overall level of reported adherence did not significantly differ, but patients in the PAC group reported that it was easier for them to adhere to the treatment recommendations (change, −2.20; 95% CI, −3.02 to −1.38; *P* < .001). Details on these parameters are presented in eTable 5 in Supplement 2. Further analyses for all available data can be found in eTables 6-8 in Supplement 2.

## Discussion

This multicenter randomized clinical trial demonstrated the additive impact of PAC on regular IIPT. PAC led to a lower pain severity and emotional impairment 3 and 6 months after discharge from IIPT compared with usual care. In line with prior studies,^3^ we found that IIPT without specialized aftercare was highly effective in reducing overall pain severity for more than 60% of usual care patients (CPG ≤2). However, the reduction was significantly greater when IIPT was followed by PAC, with 86% of these patients reaching CPG 2 or lower 6 months after discharge. The core component of the pain severity measure we used was level of functioning, of which improvement is a central goal of IIPT and of high clinical relevance.^1^ Against the background of the transtheoretical model^5^ patients receiving PAC were regularly encouraged by their social worker to continue engaging with and adapting their new strategies into everyday life. When difficulties were experienced, the social worker was available to support the patient and the family.

PAC also had a beneficial impact on emotional functioning. At 3 and 6 months after IIPT, the PAC group showed a continued reduction in anxiety and depressive symptoms. In contrast, the usual care group did not experience this improvement and instead reverted to levels of psychological symptoms experienced pre-IIPT. This corresponds with previous research^3,19^ that has found only small long-term benefits associated with IIPT in anxiety and depression. Moreover, a study by Moessner et al^20^ that evaluated the effectiveness of an aftercare program for adult patients with lower back pain also reported a reversion in posttreatment trajectories for anxiety and depression in the absence of an aftercare program.

Results of this randomized clinical trial illustrate that the patients’ pain self-efficacy was improved by PAC. In contrast, pain self-efficacy in usual care patients decreased again after discharge, although it remained higher than pre-IIPT levels. According to the theory of self-efficacy by Bandura,^21^ there are 4 sources of efficacy: mastery experiences, vicarious experiences, verbal persuasion, and emotional state. Most of these are likely to have been addressed by PAC. For example, the social workers may have facilitated more mastery experiences by motivating patients to continue with their normal daily activities despite pain. An increase in self-efficacy beliefs likely contributed to the improvement in functioning and emotional well-being observed in the PAC group, as supported by previous work.^15^

Interestingly, no significant correlation was found between frequency or intensity of PAC and its effectiveness. During PAC, patients determined the frequency of contact with the social worker, which aligns with the “What you need is what you get” design advised for patient-controlled analgesia studies.^22^ This design component allowed PAC to respond to the individual needs of patients.

### Limitations

This study has some limitations. As an inherent drawback of real-world trials, no detailed insight into the treatment’s possible modes of action can be drawn. Thus, mechanisms underlying the positive effects of PAC remain unclear. Furthermore, the high dropout rate might have resulted in a selection bias. Moreover, this study lacks a follow-up assessment after the completion of PAC. Therefore, it remains unclear whether and how long the positive effects of PAC can be maintained. Additionally, the findings may not be generalizable to other countries.

## Conclusions

This randomized clinical trial is the first study, to our knowledge, to investigate and find robust support for the effectiveness of a PAC program for pediatric patients receiving IIPT. Future research should investigate the modes of action and the long-term effects of the program.

## Notes

Supplement 1.
Trial Protocol

Supplement 2. **eAppendix 1.** Intensive Interdisciplinary Pain Treatment **eAppendix 2.** Detailed Description of the Personalized Psychosocial Aftercare Intervention **eAppendix 3.** Details on Data Analyses **eAppendix 4.** Missing Data Analyses **eTable 1.** Characteristics of the Psychosocial Aftercare Program **eTable 2.** Sociodemographic and Pain-Related Characteristics at Study Inclusion of Participants Who Did Note Dropout and Those Who Did **eTable 3.** Distribution of Chronic Pain Grading Levels at Pre-IIPT, 3 Months, and 6 Months **eTable 4.** Distribution of CPG Levels at Pre-IIPT, 3 Months, and 6 Months for All Available Data **eTable 5.** Treatment Satisfaction and Adherence **eTable 6.** Secondary Outcomes Based on All Available Data **eTable 7.** Secondary Outcomes After Multiple Imputation **eTable 8.** Mixed-Model Analyses Based on All Available Data **eFigure.** Phases of the Psychosocial Aftercare Program
eReferences.

Supplement 3.
Data Sharing Statement
