# Supplementary material for: Effectiveness of a Psychosocial Aftercare Program for Youth Aged 8 to 17 Years With Severe Chronic Pain: A Randomized Clinical Trial
Source: JAMA Netw Open. 2021 Sep 27;4(9):e2127024. doi: 10.1001/jamanetworkopen.2021.27024 (PMC8477265; doi:10.1001/jamanetworkopen.2021.27024)
Supplement: Supplement 1. — Trial Protocol [file jamanetwopen-e2127024-s001.pdf]

Supplementary Material 1. Study protocol and statistical analysis plan

DRKS-ID: **DRKS00015230**

Date of Registration in DRKS: **2018/08/06**

Date of Registration in Partner Registry or other Primary Registry: **[---]***

Deutsches Register Klinischer Studien
German Clinical Trials Register

Trial Description

Title

Socio-medical aftercare for severely chronified pediatric pain patients: A randomized controlled multicenter study

Trial Acronym

SCHMERZ-NETZ

URL of the trial

[---]*

Brief Summary in Lay Language

For children and adolescents with severe chronic pain, multimodal interdisciplinary inpatient pain therapy is the most effective form of treatment. After discharge, however, patients and their families enter a critical phase and have to apply the lessons learned in during treatment into their everyday life. The aim of this intervention study is to transfer the concept of social medical aftercare to this group of patients: after discharge, the family will be accompanied by a case manager/social worker for another 3 to 6 months. The primary goal here is help for self-help. It is to be demonstrated that this form of aftercare is effective for young pain patients: it is expected that patients who receive aftercare will show better pain-rlated and emotional outcomes one year after the inpatient treatment than the patients who do not receive it.

Brief Summary in Scientific Language

According to conservative estimates, 350,000 children and adolescents in Germany (3% to 5% of this age group) suffer from severely debilitating, disabling chronic pain. The currently best therapy for severely debilitating, disabling chronic pain is an interdisciplinary multi-modal pain therapy. In Germany there are currently three specialised children's pain centres (Datteln, Stuttgart and Augsburg), all of which work according to the same interdisciplinary multi-modal therapy approach and offer a three-week stationary therapy. However, there is a critical phase for all patients after stationary discharge, which is crucial for the long-term treatment progress. The inpatient pain therapy is short and intensive, the patients learn a lot of new things, which they then have to transfer into their everyday life, and they receive a multitude of recommendations for further outpatient treatment. A personalized socio medical aftercare (SMN), which is specifically tailored to the needs of patients, is aimed at ensuring a smooth return to everyday life. SMN offers, above all, support in the implementation and coordination of recommended services and outpatient offers. In the treatment of premature and risk-born babies, SMN has already proved tob e effective in a prospective randomized study, measuring the burden of the affected children and families as well as the number of inpatient hospital stays and outpatient emergency treatments. (Porz et al., 2006). In addition, a metaanalysis has demonstrated that adherence supporting interventions for children and adolescents with chronic diseases improve their self-management, and also reduce the severity of the disease. Although SMN is intended for chronically ill or seriously ill children

and adolescents, it is not yet used in the treatment of the group of patients with severely debilitating, disabling chronic pain. However, it is supposed that this group of patients would also benefit greatly from a SMN. For the evaluation of the new socio-medical aftercare programme, a multicenter randomized controlled study design is carried out with five measuring points (inpatient admission, discharge, 3-, 6-and 12-month follow-up). Both study groups receive the usual aftercare, since pain therapy with the current aftercare is already effective for 60% of patients and no patient is to be deprived of effective therapy. The intervention group also receives SMN. The children and adolescents are recruited consecutively at inpatient admission to the pain ward of one of the three participating clinics (Children's and Adolescents' Hospital Datteln, Olgaspital Stuttgart or Klinikum Augsburg). The reason for presentation is complex persistent pain. In addition to the patient, the parents are also asked to participate in the study. SMN is expected to result in a better treatment outcome. Another data source is the routine data of the participating health insurance companies. This includes data from inpatient stays, outpatient medical contacts, outpatient diagnoses, prescription data and medicines ordinances (Heilmittelverordnungen) and resource regulations (Hilfsmittelverordnungen). Data are extracted 3 years before inpatient admission and 3 years after discharge. In the light of current events, patients are additionally asked for data on their experience of the corona pandemic as part of the 12-month follow-up. In addition to the primary objective of the study, this should provide insights into the effects of the corona pandemic on this specific group of patients.

Do you plan to share individual participant data with other researchers?

[---]*

Description IPD sharing plan

[---]*

Organizational Data

- DRKS-ID: **DRKS00015230**
- Date of Registration in DRKS: **2018/08/06**
- Date of Registration in Partner Registry or other Primary Registry: **[---]***
- Investigator Sponsored/Initiated Trial (IST/IIT): **yes**
- Ethics Approval/Approval of the Ethics Committee: **Approved**
- (leading) Ethics Committee Nr.: **89/2018 , Ethik-Kommission der Universität Witten/Herdecke**

Secondary IDs

Health condition or Problem studied

- ICD10: **F45.4 - Persistent somatoform pain disorder**

Interventions/Observational Groups

- Arm 1: **Pediatric pain patients with usual aftercare after discharge from inpatient treatment (TAU).**
- Arm 2: **Pediatric pain patients with social-medical aftercare after discharge from inpatient treatment (SMN)**

Characteristics

- Study Type: **Interventional**
- Study Type Non-Interventional: **[---]***
- Allocation: **Randomized controlled trial**
- Blinding: **[---]***
- Who is blinded: **[---]***
- Control: **Active control (effective treament of control group)**
- Purpose: **Treatment**
- Assignment: **Parallel**
- Phase: **N/A**
- Off-label use (Zulassungsüberschreitende Anwendung eines Arzneimittels): **N/A**

Primary Outcome

Chronic pain grading: It is assumed that the patients in the SMN group show a lower pain severity 6 months after discharge, i.e. show lower CPG levels than the control group. (Note: Compared to the 1st version of the study document, the primary endpoint has been shifted from 12-month follow-up to 6-month follow-up. This adjustment was necessary due to the corona pandemic, since the data collection for the 12-month follow-up (March 2020 to December 2020) falls within the period of corona pandemic-related school closures and therefore the parameter "pain-related school absence" used in the CPG calculation cannot be validly captured.)

Secondary Outcome

Secondary endpoints: The following hypotheses are formulated for the secondary endpoints:

• Pain characteristics: At all follow-ups, children and adolescents in the intervention group SMN pain show a greater reduction in pain intensity, pain-related impairment in everyday life and pain-related school days as patients of the control group.

• Emotional impairment: At all follow-ups, children and adolescents in the

intervention group show a greater reduction of anxiety and depression and a greater increase in the quality of life than patients in the control group.

• Therapy adherence: Children and adolescents in the intervention group have implemented therapy recommendations more frequently at all follow-ups than patients in the control group.

• Therapy satisfaction: Children and adolescents in the intervention group show greater treatment satisfaction at all follow-ups than patients in the control group.
• Use of health services: in the year after inpatient treatment, patients in the intervention group are more likely to take advantage of the health benefits recommended when dismissed than patients in the control group.
• Routine data: In the year following the inpatient treatment, both patient groups cause comparable costs. In subsequent years, the costs incurred by the intervention group are below those of the control group.
• Effects of the corona pandemic: At the 12-month follow-up, children and adolescents in the intervention group show a lower emotional burden resulting from the corona pandemic than patients in the control group; additional exploratory analyses planned

Countries of recruitment

- DE **Germany**

Locations of Recruitment

- Medical Center **Vestische Kinder- und Jugendklinik, Datteln**
- Medical Center **Klinikum Stuttgart – Olgahospital, Stuttgart**
- Medical Center **Klinikum Augsburg, Augsburg**

Recruitment

- Planned/Actual: **Actual**
- (Anticipated or Actual) Date of First Enrollment: **2018/09/01**
- Target Sample Size: **394**
- Monocenter/Multicenter trial: **Multicenter trial**
- National/International: **National**

Inclusion Criteria

- Gender: **Both, male and female**
- Minimum Age: **8 Years**
- Maximum Age: **17 Years**

Additional Inclusion Criteria

1. Complex persistent pain.

2. An age-appropriate German language and reading comprehension to answer the questionnaires
3. Initial admission to pain station ward the study period
4. Consent for the participation of the parent and the patient

Exclusion criteria

No consent to participate

Addresses

- **Primary Sponsor**

Universität Witten/Herdecke
58448 Witten
Germany

Telephone: **[---]***
Fax: **[---]***
E-mail: **[---]***
URL: **[---]***

- **Contact for Scientific Queries**

Vestische Kinder- und Jugendklinik Datteln Universität Witten/ HerdeckeDeutsches Kinderschmerzzentrum
Ms. Meltem Dogan
Dr.-Friedrich-Steiner-Straße 5
45711 Datteln
Germany

Telephone: **0049-2363-975-8062**
Fax: **[---]***
E-mail: **m.dogan at deutsches-kinderschmerzzentrum.de**
URL: **[---]***

- **Contact for Public Queries**

Vestische Kinder- und JugendklinikUniversität Witten/HerdeckeDeutsches Kinderschmerzzentrum
Ms. Meltem Dogan
Dr.-Friedrich-Steiner-Str. 5
45711 Datteln
Germany

Telephone: **0049-2363-975-8062**
Fax: **[---]***
E-mail: **m.dogan at deutsches-kinderschmerzzentrum.de**
URL: **[---]***

Sources of Monetary or Material Support

- **Public funding institutions financed by tax money/Government funding body (German Research Foundation (DFG), Federal Ministry of Education and Research (BMBF), etc.)**

  Innovationsausschuss beim Gemeinsamen Bundesausschuss
  10623 Berlin
  Germany

  Telephone: **[---]***
  Fax: **[---]***
  E-mail: **[---]***
  URL: **[---]***

Status

- Recruitment Status: **Recruiting complete, follow-up continuing**
- Study Closing (LPLV): **[---]***

Trial Publications, Results and other documents

** This entry means the parameter is not applicable or has not been set.*

**** This entry means that data is not displayed due to insufficient data privacy clearing.*

Original Statistical Analysis Plan:

Primary Outcome: Mann-Whitney-U test with intervention condition as an independent variable and the Chronic Pain Grading (CPG; ordinal scale level) at 6-MONTHS as a dependent variable.

Secondary Outcomes: To study the course of the other parameters, structural equation models are used because they allow to model the modification of different variables at the same time. In particular, two Latent Growth Models are estimated, one for variables of emotional impairment (anxiety, depression, quality of life) and the other for the individual parameters that are integrated into the CPG (pain intensity, pain-related impairment, school absence).

Differences in therapy satisfaction and adherence to therapy between the intervention conditions at the measuring time (3-MONTHS, 6- MONTHS) are evaluated with t-tests for independent samples and quantified on the basis of effect sizes.

In order to determine which patients in the intervention group have a particularly good outcome or still have a negative outcome in the control group, two separate logistic regressions are performed. These each use therapy success as a criterion (success = CPG 0 or 1; no success = CPG 2, 3, or 4) and various demographic and psychosocial characteristics (e.g. gender, age, socioeconomic status, stress factors in the family) as predictors.

The significance level for multiple tests on a question is corrected according to Bonferroni-Holm (Holm 1979).

Sample calculation: The primary endpoint of the study is the difference between the intervention and control group with respect to the CPG for the 12-month follow-up. This difference is evaluated using the Mann-Whitney-U test. With an alpha level of 5% and a power of 95%, N=184 children (n=92 per group) must be included in the analyses to identify a medium effect (d=0.5). If a 7% discontinuation of therapy and discontinuation of studies are assumed in 20% of patients and a dropout rate of 30% at the 12-month follow-up (comparable to previous studies of the DKSZ), a total sample of N=394 (patients randomized) results.

Changes to the Statistical Analysis Plan:

The original analysis plan specified differences in CPG levels at 12-MONTHS as the primary endpoint. At the start of 2020 it became apparent that the COVID-19 pandemic would exert a huge impact on our study. In particular, the first lockdown and closure of schools in Germany made it necessary to change the planned analysis because central variables such as school absence and certain forms of pain-related disability could no longer be reliably assessed. We thus decided to use 6-MONTHS CPG as the primary endpoint.

The original analysis plan also specified more in-depth analysis of the secondary outcomes using longitudinal latent growth models. These, too, were affected by the compromised 12-MONTHS follow-up. As the lack of this additional data point restricts what models can be fit, we decided to use a less complex mixed-model analysis to characterize the changes in the individual secondary outcome variables. However, we have applied for a grant to collect additional data in this sample and will try to investigate development over time – including COVID-19 related stress – in a different publication.
