# Supplementary material for: Effectiveness of a Psychosocial Aftercare Program for Youth Aged 8 to 17 Years With Severe Chronic Pain: A Randomized Clinical Trial
Source: JAMA Netw Open. 2021 Sep 27;4(9):e2127024. doi: 10.1001/jamanetworkopen.2021.27024 (PMC8477265; doi:10.1001/jamanetworkopen.2021.27024)
Supplement: Supplement 2. — eAppendix 1. Intensive Interdisciplinary Pain Treatment eAppendix 2. Detailed Description of the Personalized Psychosocial Aftercare Intervention eAppendix 3. Details on Data Analyses eAppendix 4. Missing Data Analyses eTable 1. Characteristics of the Psychosocial Aftercare Program eTable 2. Sociodemographic and Pain-Related Characteristics at Study Inclusion of Participants Who Did Note Dropout and Those Who Did eTable 3. Distribution of Chronic Pain Grading Levels at Pre-IIPT, 3 Months, and 6 Months eTable 4. Distribution of CPG Levels at Pre-IIPT, 3 Months, and 6 Months for All Available Data eTable 5. Treatment Satisfaction and Adherence eTable 6. Secondary Outcomes Based on All Available Data eTable 7. Secondary Outcomes After Multiple Imputation eTable 8. Mixed-Model Analyses Based on All Available Data eFigure. Phases of the Psychosocial Aftercare Program eReferences. [file jamanetwopen-e2127024-s002.pdf]

Supplemental Online Content

Dogan M, Hirschfeld G, Blankenburg M, et al. Effectiveness of a psychosocial aftercare program for youth aged 8 to 17 years with severe chronic pain: a randomized clinical trial. *JAMA Netw Open*. 2021;4(9):e2127024. doi:10.1001/jamanetworkopen.2021.27024

eAppendix 1. Intensive Interdisciplinary Pain Treatment
eAppendix 2. Detailed Description of the Personalized Psychosocial Aftercare Intervention
eAppendix 3. Details on Data Analyses
eAppendix 4. Missing Data Analyses
eTable 1. Characteristics of the Psychosocial Aftercare Program
eTable 2. Sociodemographic and Pain-Related Characteristics at Study Inclusion of Participants Who Did Note Dropout and Those Who Did
eTable 3. Distribution of Chronic Pain Grading Levels at Pre-IIPT, 3 Months, and 6 Months
eTable 4. Distribution of CPG Levels at Pre-IIPT, 3 Months, and 6 Months for All Available Data
eTable 5. Treatment Satisfaction and Adherence
eTable 6. Secondary Outcomes Based on All Available Data
eTable 7. Secondary Outcomes After Multiple Imputation
eTable 8. Mixed-Model Analyses Based on All Available Data
eFigure. Phases of the Psychosocial Aftercare Program
eReferences

This supplemental material has been provided by the authors to give readers additional information about their work.

eAppendix 1. Intensive Interdisciplinary Pain Treatment

The intensive interdisciplinary pain treatment (IIPT) applied in this study followed a treatment manual [4]. Patients are admitted to a chronic pain unit for 3 to 4 weeks and receive intensive treatment for about 8 hours a day. Treatment is delivered by an interdisciplinary team of pediatricians, clinical psychologists, pediatric nurses, physiotherapists, occupational therapists, and social workers [7]. The treatment approach combines the following six components: 1) pain education and realistic goal setting; 2) training on pain-coping strategies; 3) treatment of co-occurring emotional distress; 4) family therapy; 5) optional therapy-related drug treatment or physiotherapy; and 6) relapse prevention [4]. Within this treatment approach, the patients' families are heavily involved. Like the patient, they receive pain-related education to address dysfunctional parental cognitions and maladaptive behavioral patterns. Concurrently strengthening the parents' abilities to cope with their child's pain is a primary focus [4]. Family sessions take place once a week.

At discharge from IIPT, patients and their families are given a personalized discharge plan containing specific recommendations, such as ensuring regular school attendance and seeking outpatient psychotherapy. Furthermore, all patients are recommended to return to the pain center three and six months after discharge for follow-up meetings with the treating pediatrician and the psychotherapist from IIPT. The aim of these appointments is to give patients the opportunity to evaluate his or her treatment progress and clarify any questions.

© 2021 Dogan M et al. *JAMA Network Open.*

eAppendix 2. Detailed Description of the Personalized Psychosocial Aftercare Intervention

Personalized psychosocial aftercare (PAC) is based on a standardized manual, following the model of the German "sociomedical aftercare" treatment approach and guided by the concept of case management [9,13]. This methodology is used in other chronic health fields and aims to provide a roadmap for improving patient-well-being via close collaboration with the patient and their family. This family-centered care approach is generally implemented following inpatient treatment as collaborative and often interdisciplinary process. During treatment, therapeutic or social services and coping strategies to enhance the patient's health are assessed, planed, coordinated, implemented, monitored and evaluated. Pediatric pain experts at the German Pediatric Pain Centre, Children's and Adolescents' Hospital Datteln noted this treatment approach for its potential utility to maintain treatment gains established during intensive interdisciplinary pain treatment (IIPT) and adapted to the needs of young people with chronic pain. PAC was piloted and updated based on feedback from staff and patients prior to the commencement of the randomized controlled trial (not published).

The aims of PAC were to enable and motivate patients and their familes to implement their personalized discharge plan in their everyday lives, providing needs-oriented professional support, promoting positive health behaviors and strengthening the functioning of the patient and the family system. The phases of PAC can be seen in **eFigure 1**. The mode, content, frequency and intensity of PAC were implemented in a need-orientated and individualized way for each patient and their family, comparable to the WYNIWYG (what you need is what you get) concept known in patient-controlled analgesia [10].

In summary, for each IIPT patient in the intervention group, the PAC intervention commenced at the IIPT discharge meeting in which the PAC social worker was introduced to patients and their family. During this session, the PAC social worker arranged an introductory phone call with the family one week after discharge. In the first phone-based contact, the social worker assessed treatment expectations and collaborated with the patients and families to set goals for future clinical contact. Some families requested weekly appointments, discussing their pain-related or everyday problems with the social worker, while others requested support for a single issue, such as finding an outpatient psychotherapist. For the average PAC frequency and intensity see **eTable 1**. Patients and their parents were able to call or text the PAC team between scheduled appointments if support or consultation was needed. After five to six weeks of aftercare, all families were offered a home visit by the social worker (irrelevant of the distance from the hospital to their home), to further build rapport as well as to identify environmental and other barriers to treatment outcomes. Contact with the PAC social worker was mostly via phone, but occasionally via email, video calls and home visits. PAC continued for up to six months as requested by the participants and their families. Throughout the PAC process, the social worker observed and recorded the progress of goal attainment according to the discharge plan and individual expectations voiced at the beginning of PAC. By empowering the families and enhancing patient self-efficacy through positive affirmations or accentuating mastery experiences [1], motivation and optimism increased. While achievements did not necessarily indicate that a patient and their family no longer required support, it demonstrated that a family system could successfully implement the discharge plan mutually planned with the social worker, the patient and their family.

To guarantee that IIPT and PAC staff were sufficiently familiarized with the concept of PAC, the intervention was piloted and adapted based on feedback. During the intervention, PAC social workers worked closely with the physicians and psychologists involved in the IIPT and received detailed briefings at the commencement of the intervention. Furthermore, a consistent PAC-procedure was set through regular meetings with all three study centers. The social worker at the main study center was involved in the program conceptualization and provided continuous supervision throughout.

The PAC manual (in German) can be requested from the corresponding author.

eAppendix 3. Details on Data Analyses

The primary endpoint of our study was patient pain severity at 6-MONTHS. In order to classify pain severity, we used the validated **Chronic Pain Grading (CPG)** according to Wager et al. [14]. Children and adolescents who had not reported chronic pain (i.e., permanent or recurrent pain in the past three months) were classified as CPG 0 (no chronic pain). For the other four CPG categories, data on the patient‘s pain intensity, missed school days due to pain as well as pain-related disability in everyday life (as measured by the Paediatric Pain Disability Inventory; PPDI [6]) were integrated using an algorithm. The mean of average and maximal pain intensity within the last four weeks was categorized as low (< 5 / 10) or high (≥ 5 / 10). Moreover, patients were assigned disability points according to their PPDI summed scores (0 = 12 - 27, 1 = 28 - 35, 2 = 36 - 42, 3 = 43 - 60) and their number of days absent from school (0 = none, 1 = one, 2 = two to five, 3 = more than five). Then, the sum of these disability points was calculated, thus allocating each patient with chronic pain to one of the following three disability categories: 0 – 2 = low disability, 3 – 4 = moderately limiting, 5 – 6 = severely limiting.
Based on pain intensity and pain disability, pain severity grades 1 - 4 were specified as follows:

- CPG 1 = low disability (0 - 2 disability points) and low pain intensity (< 5 / 10);
- CPG 2 = low disability (0 - 2 disability points) and high pain intensity (≥ 5 / 10);
- CPG 3 = high disability, moderately limiting (3 - 4 disability points);
- CPG 4 = high disability, severely limiting (5 - 6 disability points).

Changes in the primary outcome CPG from PRE-IIPT to 3- and 6-MONTHS were calculated using the Wilcoxon test. Pain outcomes at 3- and 6-MONTHS were compared to PRE-IIPT scores because disability scores were not calculable at POST-IIPT. We then used the Mann-Whitney-U test to compare the distribution of the primary outcome (CPG) between the two groups at 3- and 6-MONTHS. Effect sizes for Wilcoxon tests were calculated as r = z/ sqrt(N), and interpreted according to established conventions [3]. Confidence intervals (95%) for the effect sizes were constructed using bootstrapping.

For the analyses of our secondary outcome variables (all continuous data), we used a mixed-model framework to fit separate conditional growth models for each outcome. These models used time, group and the time*group interaction as fixed effects and included random intercepts for patients and random effects for time, group and their interaction for the different study sites. Furthermore, a simple autocorrelation (AR1) term was added to account for correlations between measurements from one patient. Within these models, time was treated as a categorical predictor with the PRE-IIPT time point serving as the reference category. Group was also treated as a categorical predictor with TAU(control group) as the reference category. As such, effects for assessment time point may be interpreted as the difference between PRE-IIPT and either POST-IIPT, 3-MONTHS and 6-MONTHS, and effects for group are differences between TAU and PAC. The main coefficients of interest were the time*group interactions since these indicate differences in how the two groups change over time. Main effects for time and group were included because both groups received an active treatment at the beginning of the trial and thus we expected changes related to time. Group was included in case there were systematic differences between the groups after randomization.

As indicated in the flowchart (Figure 1) we had considerable dropout rates due to COVID-19 and other reasons. The tables and figures reported in the main body are based on the *n*=222 patients who supplied data at 6-MONTHS, including those patients who did not respond to the POST-IIPT or 3-MONTHS assessments. There were *n*=12 patients who did not respond to questionnaires at POST-IIPT and *n*=15 who did not respond at 3-MONTHS. There were no systematic differences in the missingness between the groups regarding demographic or pain-related variables as tested with chi-square and Welch tests (eTable 2). We ran several analyses with and without those patients who supplied partial information, and with and without multiple imputation and found no substantial differences in the results. The results of the mixed-model analyses after multiple imputation are shown in eTable 8.

All analyses were performed using R (Version 4.0.3) and the packages coin [5] for wilcoxon tests, nlme [12] for mixed models and mice [2] for multiple imputation.

Missing data was prevented by collecting data electronically using force-choice programmed surveys.

© 2021 Dogan M et al. *JAMA Network Open.*

eAppendix 4. Missing Data Analyses

As a sensitivity analysis we ran several additional analyses based on all available data and with and without multiple imputation and found no substantial differences in the results.

Missing data analyses for the primary outcome CPG

eTable 5 shows the CPG levels at the assessment time points based on all available data. Within this dataset we used two different approaches to test for group differences at the three time points: all available data and multiple imputation. For multiple imputation we used 20 multiple imputations and simple sampling as the imputation method. There were no missing data at PRE-IIPT, while 25% missing data were imputed at 3-MONTHS and 47% missing data were imputed at 6-MONTHS. Since there is some debate about the proper pooling of results from non-parametric tests [8], we used a procedure suggested by Licht [11] to pool the results from the different imputed datasets. This involves transforming p-values to z-scores, which in turn are averaged and transformed back to p-values. The median p-value is the reported result.
Using all available data, we found a significant moderate difference between the two groups at 6-MONTHS (Z = 4.50; r = 0.30; 95% CI = 0.18 – 0.42; p <.001) and a small effect at 3-MONTHS (Z = 2.94; r = 0.17; 95% CI = 0.03 – 0.27; p =.002) but not at PRE-IIPT (Z = -0.88; r = 0.04; p = .81). These analyses agree with the analyses of the n=222 patients and shows that there are significant differences in the pain severity at 3-MONTHS and 6-MONTHS.
The results after multiple imputation yield similar results. Specifically, we find a significant difference between the groups at 6-MONTHS (*p*Licht = .002; *p*Median <.001), and at 3-MONTHS (*p*Licht = .015; *p*Median =.008). No data were imputed for PRE-IIPT.

Missing data analyses for secondary outcomes

eTable 6 shows the descriptive statistics for the secondary outcomes based on all available data. In this dataset, no data were missing at PRE-IIPT, 16% were missing at POST-IIPT, 25% were missing at 3-MONTHS and 47% were missing at 6-MONTHS. We ran two different analyses on this dataset: multiple imputation for t-tests and all available data for mixed models. The multiple imputation method involved 20 imputations (method: partial mean matching), performing a simple linear regression (value predicted by group) and pooling the results of the group coefficient. Results of the group comparisons after multiple imputation are shown in eTable 7. Again, the multiple imputation analysis agrees with the analysis of the *n*=222 patients and shows that there are significant differences in the pain-related and psychological variables that emerge at 3-MONTHS and 6-MONTHS.
We decided not to use multiple imputation for the mixed model analysis because an analysis based on all available data is as efficient as multiple imputation. The results of these analyses are shown in eTable 8. These also agree with the analysis based on the *n*=222 patients who supplied data at 6-MONTHS.

© 2021 Dogan M et al. *JAMA Network Open.*

eTable 1. Characteristics of the Psychosocial Aftercare Program

**Mode of contact**	**Frequency of contact per patient**<br>M (SD)<br>[range]	**Duration of contact (min.)**<br>M (SD)<br>[range]
Phone calls	14.9 (3.1)<br>[1-35]	12.5 (8.9)<br>[3-70]
Video calls	0.1 (0.1)<br>[0-12]	31.5 (13.7)<br>[14-75]
E-mails [a]	0.5 (0.3)<br>[0-18]	4.7 (0.4)<br>[1-20]
Home visits	0.3 (0.1)<br>[0-2]	116.2 (7.1)<br>[60-230]
External meetings (e.g., accompaniment to doctor appointments)	1.1 (0.1)<br>[0-2]	41.7 (8.3)<br>[25-60]

[a] Outgoing e-mails written by social workers

© 2021 Dogan M et al. *JAMA Network Open.*

eTable 2. Sociodemographic and Pain-Related Characteristics at Study Inclusion of Participants Who Did Note Dropout and Those Who Did

	**Non-dropouts (1)**	**Dropouts related to COVID-19 (2)**	**Dropouts for other reasons (3)**	***p*-value overall**	***p*-value (1)vs.(2)**	***p*-value (1)vs.(3)**	***p*-value (2)vs.(3)**
	n=222	***n=102***	***n=95***				
Sex				.817	.886	.886	.886
Female	160 (72.1%)	76 (74.5%)	67 (70.5%)				
Male	62 (27.9%)	26 (25.5%)	28 (29.5%)				
Age	14.1 (2.20)	14.5 (1.95)	14.6 (1.92)	.060	.211	.088	.914
Pain locations[a]				.562	.533	.642	.524
Head	153 (68.9%)	66 (64.7%)	69 (72.6%)				
Abdomen	60 (27.0%)	23 (22.5%)	26 (27.4%)				
Musculoskeletal	93 (41.9%)	44 (43.1%)	46 (48.4%)				
> 1 main pain location	68 (30.8%)	28 (27.5%)	30 (31.6%)				
Pain duration				.669	.743	.772	.743

© 2021 Dogan M et al. *JAMA Network Open.*

	Non-dropouts (1)	Dropouts related to COVID-19 (2)	Dropouts for other reasons (3)	*p*-value overall	*p*-value (1)vs.(2)	*p*-value (1)vs.(3)	*p*-value (2)vs.(3)
	n=222	***n=102***	***n=95***				
3-6 months	21 (9.5%)	11 (10.8%)	5 (5.3%)				
6-12 months	48 (21.6%)	15 (14.7%)	22 (23.2%)				
1-2 years	37 (16.7%)	15 (14.7%)	17 (17.9%)				
2-3 year	43 (19.4%)	19 (18.6%)	17 (17.9%)				
More than 3 years	73 (32.9%)	42 (41.2%)	34 (35.8%)				
Maximum pain intensity [b]	8.17 (1.65)	8.17 (1.72)	8.07 (1.97)	.894	1.000	.892	.926
Average pain intensity [b]	6.14 (1.83)	5.77 (1.95)	6.22 (1.96)	.187	.249	.927	.223
Missed school days [b]	5.67 (6.86)	4.44 (6.49)	5.99 (7.52)	.229	.302	.923	.261
Pain severity (CPG)[c]				.185	.222	.641	.343

© 2021 Dogan M et al. *JAMA Network Open.*

	Non-dropouts (1)	Dropouts related to COVID-19 (2)	Dropouts for other reasons (3)	*p*-value overall	*p*-value (1)vs.(2)	*p*-value (1)vs.(3)	*p*-value (2)vs.(3)
	n=222	***n=102***	***n=95***				
1	15 (6.8%)	9 (8.8%)	7 (7.4%)				
2	68 (30.6%)	42 (41.2%)	27 (28.4%)				
3	69 (31.1%)	32 (31.4%)	36 (37.9%)				
4	70 (31.5%)	19 (18.6%)	25 (25.3%)				

[a] Multiple pain locations could be selected, [b] In the past 4 weeks, [c] No participants had a Chronic Pain Grading (CPG) score of 0 at admission.

© 2021 Dogan M et al. *JAMA Network Open.*

eTable 3. Distribution of Chronic Pain Grading Levels at Pre-IIPT, 3 Months, and 6 Months

	**PRE-IIPT**		**3-MONTHS**		**6-MONTHS**	
	**TAU**	**PAC**	**TAU**	**PAC**	**TAU**	**PAC**
	N=107	***N=115***	***N=99***	***N=108***	***N=107***	***N=115***
Pain severity (CPG level)	Median=3 IQR=2-4	Median=3 IQR=2-4	Median=2 IQR=2-3	Median=2 IQR=1-3	Median=2 IQR=2-3	Median=1 IQR=1-2
0	0 (0.0%)	0 (0.0%)	8 (8.1%)	12 (11.1%)	10 (9.3%)	20 (17.4%)
1	8 (7.5%)	7 (6.1%)	10 (10.1%)	27 (25.0%)	15 (14.0%)	38 (33.0%)
2	37 (34.6%)	31 (27.0%)	47 (47.5%)	40 (37.0%)	43 (40.2%)	41 (35.7%)
3	33 (30.8%)	36 (31.3%)	14 (14.1%)	17 (15.7%)	23 (21.5%)	9 (7.8%)
4	29 (27.1%)	41 (35.7%)	20 (20.2%)	12 (11.1%)	16 (15.0%)	7 (6.1%)

Note: Missed school days and pain-related disability were not assessable at POST-IIPT; TAU = treatment as usual; PAC = psychosocial aftercare; CPG = Chronic Pain Grading

© 2021 Dogan M et al. *JAMA Network Open.*

eTable 4. Distribution of CPG Levels at Pre-IIPT, 3 Months, and 6 Months for All Available Data

	PRE-IIPT		3-MONTHS		6-MONTHS	
	**TAU**	**PAC**	**TAU**	**PAC**	**TAU**	**PAC**
	N=218	***N=201***	***N=161***	***N=152***	***N=107***	***N=115***
Pain severity (CPG level)	Median=3 IQR=2-4	Median=3 IQR=2-4	Median=2 IQR=2-3	Median=2 IQR=1-3	Median=2 IQR=2-3	Median=1 IQR=1-2
0	0 (0.0%)	0 (0.0%)	15 (9.3%)	18 (11.8%)	10 (9.3%)	20 (17.4%)
1	16 (7.3%)	15 (7.5%)	18 (11.2%)	37 (24.3%)	15 (14.0%)	38 (33.0%)
2	76 (34.9%)	61 (30.3%)	68 (42.2%)	55 (36.2%)	43 (40.2%)	41 (35.7%)
3	71 (32.6%)	66 (32.8%)	26 (16.1%)	26 (17.1%)	23 (21.5%)	9 (7.8%)
4	55 (25.2%)	59 (29.4%)	34 (21.1%)	16 (10.5%)	16 (15.0%)	7 (6.1%)

Note: Missed school days and pain-related disability were not assessable at POST-IIPT; TAU = treatment as usual; PAC = psychosocial aftercare; CPG = Chronic Pain Grading

eTable 5. Treatment Satisfaction and Adherence

	TAU	PAC	Difference	95% CI	*p*-value
Overall treatment satisfaction					
POST-IIPT	7.70 (2.22)	7.43 (2.51)	0.27	0.38 – 0.91	.417
3-MONTHS	6.25 (2.77)	7.03 (2.92)	-0.78	-1.56 – 0.00	.051
6-MONTHS	6.21 (2.86)	7.38 (2.59)	-1.18	-1.90 – -0.45	.002
Therapy adherence<br>6-MONTHS	6.44 (2.74)	6.84 (2.87)	-0.40	-1.15 – -0.34	.284
Easiness of therapy adherence<br>6-MONTHS	4.19 (3.23)	6.38 (2.95)	-2.20	-3.02 – -1.38	<.001

Note: TAU = treatment as usual; PAC = psychosocial aftercare

eTable 6. Secondary Outcomes Based on All Available Data

	PRE-IIPT		POST-IIPT		3-MONTHS		6-MONTHS	
	TAU	PAC	TAU	PAC	TAU	PAC	TAU	PAC
	N=218	*N=201*	*N=186*	*N=168*	*N=161*	*N=152*	*N=107*	*N=115*
**Pain measures**								
Maximum pain intensity (last 4 weeks)	8.08 (1.88)	8.22 (1.58)	7.67 (2.11)	7.68 (2.28)	7.19 (2.86)	6.34 (3.08)	6.86 (2.84)	5.34 (3.32)
Average pain intensity (last 4 weeks)	6.01 (1.95)	6.12 (1.84)	4.65 (2.14)	4.68 (2.32)	5.07 (2.77)	4.28 (2.61)	5.25 (2.80)	3.63 (2.88)
Missed school days (last 4 weeks)	5.24 (6.79)	5.66 (7.10)	-	-	3.13 (5.18)	2.47 (5.16)	2.71 (4.63)	1.32 (3.79)
Pain-related disability	34.80 (9.73)	35.90 (9.61)	-	-	29.70 (13.30)	26.00 (12.20)	29.50 (12.70)	22.90 (11.10)
**Psychological measures**								
Pain self-efficacy	27.1 (8.45)	27.00 (9.29)	37.70 (9.34)	36.90 (9.23)	32.50 (11.60)	37.80 (9.33)	33.30 (12.10)	40.60 (9.82)
Depression	10.5 (5.44)	11.00 (5.65)	8.60 (5.19)	9.54 (5.49)	10.90 (5.68)	9.12 (5.84)	10.80 (5.79)	8.42 (5.32)
Anxiety	26.7 (17.9)	28.3 (18.5)	24.3 (17.1)	27.3 (17.7)	26.5 (17.7)	21.6 (18.3)	25.5 (18.6)	19.9 (16.7)
Health-related quality of life	96.4 (14.9)	96.2 (15.9)	-	-	95.9 (17.7)	103.0 (16.3)	95.3 (18.4)	106.0 (14.3)

Note: TAU = treatment as usual; PAC = psychosocial aftercare

© 2021 Dogan M et al. *JAMA Network Open.*

eTable 7. Secondary Outcomes After Multiple Imputation

	PRE-IIPT	POST-IIPT	3-MONTHS	6-MONTHS
**Pain measures**				
Maximum pain intensity (last 4 weeks) [a]	0.14 (-0.20 – 0.47)	0.04 (-0.41 – 0.49)	-0.74 (-1.37 – -0.11)*	-1.03 (-1.76 – -0.30)**
Average pain intensity (last 4 weeks) [a]	0.11 (-0.25 – 0.47)	0.06 (-0.41 – 0.54)	-0.65 (-1.21 – -0.08)*	-1.11 (-1.85 – -0.37)**
Missed school days (last 4 weeks)	0.42 (-0.91 – 1.76)	-	-0.24 (-1.24 – -0.75)	-0.85 (-1.69 – -0.01)*
Pain-related disability[b]	1.11 (-0.75 – 2.97)	-	-2.74 (-5.37 – -0.1)*	-5.09 (-7.64 – -2.54)***
**Psychological measures**				
Pain self-efficacy[c]	-0.03 (-1.73 – 1.68)	-0.84 (-2.74 – 1.06)	-4.17 (1.95 – 6.39)***	5.58 (3.27 – 7.90)***
Depression[d]	0.50 (-0.56 – 1.57)	0.94 (-0.10 – 1.98)	-1.34 (-2.52 – -0.16)*	-1.70 (-2.91 – -0.49)**
Anxiety[e]	1.64 (-1.85 – 5.14)	3.45 (-0.08 – 6.99)	-3.54 (-7.17 – -0.09)	-4.33 (-8.10 – -0.56)*
Health-related quality of life[f]	-0.22 (-3.18 – 2.73)	-	6.20 (2.86 – 9.54)***	8.26 (4.71 – 11.81)***

Note: * = *p* <.05; ** = *p* <.01; *** = *p* <.001

[a] Numerical Rating Scale (NRS): Range 0-10.
[b] Paediatric Pain Disability Index (P-PDI); Range: 12-60.
[c] Scale for Pain Self-Efficacy (SPaSE); Range: 0-44.
[d] Revised Child Anxiety and Depression Scale (RCADS); Range 0-30.
[e] Revised Child Anxiety and Depression Scale (RCADS); Range 0-111.
[f] Kidscreen-27; Range: 27-135.

© 2021 Dogan M et al. *JAMA Network Open.*

eTable 8. Mixed-Model Analyses Based on All Available Data

Measure	Group	Time (POST-IIPT)	Time (POST-IIPT) X group	Time (3-MONTHS)	Time (3-MONTHS) X group	Time (6-MONTHS)	Time (6-MONTHS) X group
Maximum pain intensity	0.17 (-0.36 - 0.70)	-0.34 (-0.77 - 0.10)	-0.16 (-0.70 - 0.38)	-0.93 (-1.37 - - 0.50)	-0.98 (-1.60 - - 0.35)	-1.28 (-1.8 - - 0.76)	-1.62 (-2.35 - - 0.89)
Average pain intensity	0.12 (-0.33 - 0.58)	-1.35 (-1.69 - - 1.02)	-0.08 (-0.57 - 0.40)	-0.98 (-1.38 - - 0.57)	-0.82 (-1.48 - - 0.16)	-0.89 (-1.37 - - 0.41)	-1.58 (-2.25 - - 0.90)
Missed school days	0.44 (-0.92 - 1.80)			-2.14 (-3.06 - - 1.21)	-0.99 (-2.32 - 0.35)	-2.71 (-3.98 - - 1.44)	-1.8 (-3.60 - - 0.01)
Pain-related disability	1.10 (-1.05 - 3.25)			-5.31 (-6.95 - - 3.67)	-4.51 (-6.86 - - 2.15)	-5.17 (-7.39 - - 2.95)	-7.64 (-10.77 - - 4.51)
Pain self-efficacy	-0.02 (-1.88 - 1.84)	10.48 (9.10 - 11.85)	-0.78 (-2.77 - 1.21)	5.64 (3.94 - 7.35)	5.23 (2.77 - 7.69)	6.30 (4.08 - 8.52)	7.07 (4.14 - 10.01)
Depression	0.61 (-0.86 - 2.07)	-1.83 (-2.51 - - 1.15)	0.40 (-0.58 - 1.37)	0.40 (-0.49 - 1.29)	-2.30 (-3.47 - - 1.13)	0.21 (-0.78 - 1.20)	-2.91 (-4.30 - - 1.52)
Anxiety	1.95 (-2.70 - 6.60)	-2.09 (-3.92 - - 0.26)	1.35 (-1.31 – 4.00)	-0.20 (-2.75 - 2.36)	-6.44 (-9.77 - - 3.11)	-0.48 (-4.75 - 3.78)	-8.85 (-12.91 - - 4.79)
Health-related quality of life	-0.23 (-3.40 - 2.93)			-0.15 (-2.56 - 2.26)	7.58 (4.13 - 11.03)	-1.01 (-4.11 - 2.09)	11.16 (6.81 - 15.52)

Note: Table shows unstandardized coefficients. The reference category for time was PRE-IIPT.

© 2021 Dogan M et al. *JAMA Network Open.*

eFigure. Phases of the Psychosocial Aftercare Program

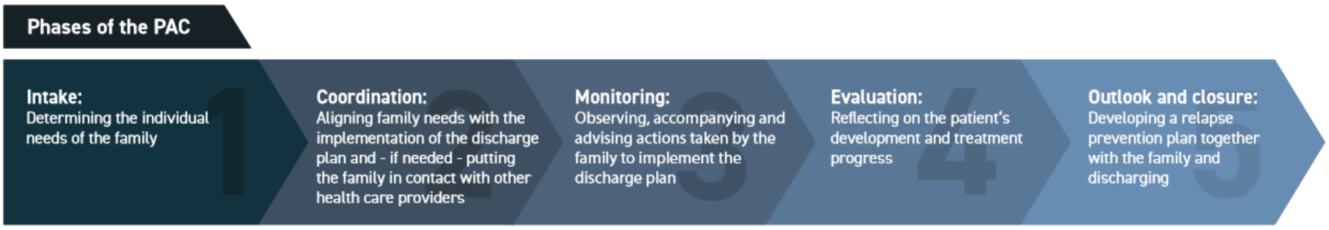

© 2021 Dogan M et al. *JAMA Network Open.*

eReferences.

[1] Bandura A. Self-efficacy: toward a unifying theory of behavioral change. Psychol Rev 1977;84(2):191-215.

[2] Buuren S, Groothuis-Oudshoorn K. mice: Multivariate Imputation by Chained Equations inR. Journal of Statistical Software. 2011;45(3).

[3] Cohen J. Statistical Power Analysis. Current Directions in Psychological Science. 1992;1(3):98-101.

[4] Dobe, M.; Zernikow, B. Practical treatment options for chronic pain in children and adolescents: An interdisciplinary therapy manual. Springer: Berlin, Heidelberg, 2019; Vol. 2, p 1-299.

[5] Hothorn T, Hornik K, Wiel M, Zeileis A. Implementing a Class of Permutation Tests: ThecoinPackage. Journal of Statistical Software. 2008;28(8).

[6] Hübner B, Hechler T, Dobe M, Damschen U, Kosfelder J, Denecke H, Schroeder S, Zernikow B. [Pain-related disability in adolescents suffering from chronic pain: Preliminary examination of the Pediatric Pain Disability Index (P-PDI)]. Schmerz 2009;23(1):20-32.

[7] IASP. Task force on multimodal pain treatment defines terms for chronic pain care. https://www.iasp-pain.org/PublicationsNews/NewsDetail.aspx?ItemNumber=6981 2017.

[8] [Internet]. Stefvanbuuren.name. 2021 [cited 18 July 2021]. Available from: https://stefvanbuuren.name/fimd/sec-multiparameter.html

[9] Kathol R, Andrew R, Squire M, Dehnel P. The integrated case management manual.

[10] Lehmann K. Recent Developments in Patient-Controlled Analgesia. Journal of Pain and Symptom Management. 2005;29(5):72-89.

[11] Licht C. New Methods for Generating Significance Levels from Multiply-Imputed Data. PhD thesis, Bamberg, Germany: University of Bamberg. 2010.

[12] Pinheiro, J, Bates, D. DebRoy, S, Sarkar, D. R Core Team (2020) nlme: linear and nonlinear mixed effects models. R package version 3.1–148. 2020.

[13] Podeswik A. Praxishandbuch Pädiatrische Nachsorge. Augsburg: Beta Instituts-Verl.; 2007.

[14] Wager, J., Hechler, T., Darlington, A. S., Hirschfeld, G., Vocks, S., & Zernikow, B. Classifying the severity of paediatric chronic pain–an application of the chronic pain grading. European journal of pain 2013; 17(9), 1393-1402.

© 2021 Dogan M et al. *JAMA Network Open.*
