# Supplementary material for: Effectiveness of a Psychosocial Aftercare Program for Youth Aged 8 to 17 Years With Severe Chronic Pain: A Randomized Clinical Trial
Source: JAMA Netw Open. 2021 Sep 27;4(9):e2127024. doi: 10.1001/jamanetworkopen.2021.27024 (PMC8477265; doi:10.1001/jamanetworkopen.2021.27024)
Supplement: Supplement 3. — Data Sharing Statement [file jamanetwopen-e2127024-s003.pdf]

Data Sharing Statement

Dogan. Effectiveness of a Psychosocial Aftercare Program for Youth Aged 8 to 17 Years With Severe Chronic Pain. *JAMA Netw Open*. Published September 27, 2021. doi:10.1001/jamanetworkopen.2021.27024

Data
Data available: No
